# Immobilized enzyme microreactors for analysis of tryptic peptides in *β*-casein and *β*-lactoglobulin

**DOI:** 10.1038/s41598-023-43521-z

**Published:** 2023-10-02

**Authors:** Agnieszka Rodzik, Viorica Railean, Paweł Pomastowski, Bogusław Buszewski, Michał Szumski

**Affiliations:** 1 Centre for Modern Interdisciplinary Technologies, Nicolaus Copernicus University in Toruń, Wileńska 4, 87-100, Toruń, Poland; 2 Department of Environmental Chemistry and Bioanalysis, Faculty of Chemistry, Nicolaus Copernicus University in Toruń, Gagarina 7, 87-100 Toruń, Poland; 3 Department of Infectious, Invasive Diseases and Veterinary Administration, Institute of Veterinary Medicine, Nicolaus Copernicus University in Torun, Gagarina 7, 87-100 Toruń, Poland

**Keywords:** Analytical chemistry, Proteins, Proteolysis, Analytical biochemistry

## Abstract

In this study, our primary objective was to develop an effective analytical method for studying trypsin-digested peptides of two proteins commonly found in cow's milk: *β*-casein (*β*CN) and *β*-lactoglobulin (*β*LG). To achieve this, we employed two distinct approaches: traditional in-gel protein digestion and protein digestion using immobilized enzyme microreactors (μ-IMER). Both methods utilized ZipTip pipette tips filled with C18 reverse phase media for sample concentration. The μ-IMER was fabricated through a multi-step process that included preconditioning the capillary, modifying its surface, synthesizing a monolithic support, and further surface modification. Its performance was evaluated under HPLC chromatography conditions using a small-molecule trypsin substrate (BAEE). Hydrolysates from both digestion methods were analyzed using MALDI-TOF MS. Our findings indicate that the μ-IMER method demonstrated superior sequence coverage for oxidized molecules in *β*CN (33 ± 1.5%) and *β*LG (65 ± 3%) compared to classical in-gel digestion (20 ± 2% for *β*CN; 49 ± 2% for *β*LG). The use of ZipTips further improved sequence coverage in both classical in-gel digestion (26 ± 1% for *β*CN; 60 ± 4% for *β*LG) and μ-IMER (41 ± 3% for *β*CN; 80 ± 5% for *β*LG). Additionally, phosphorylations were identified. For *β*CN, no phosphorylation was detected using classical digestion, but the use of ZipTips showed a value of 27 ± 4%. With μ-IMER and μ-IMER–ZipTip, the values increased to 30 ± 2% and 33 ± 1%, respectively. For *β*LG, the use of ZipTip enabled the detection of a higher percentage of modified peptides in both classical (79 ± 2%) and μ-IMER (79 ± 4%) digestions. By providing a comprehensive comparison of traditional in-gel digestion and μ-IMER methods, this study offers valuable insights into the advantages and limitations of each approach, particularly in the context of complex biological samples. The findings set a new benchmark in protein digestion and analysis, highlighting the potential of μ-IMER systems for enhanced sequence coverage and post-translational modification detection.

## Introduction

Proteins, which are abundant macromolecules in cells, are composed of linear chains of amino acids connected by peptide bonds. A valuable source of biologically active proteins is cow milk, providing essential bioactive components to the body, including high-quality proteins, lactose as a carbohydrate source, mineral salts (such as calcium and phosphorus compounds), and vitamins^1^. Milk consists of two main groups of proteins: casein (*α*_*S1*_, *α*_*S2*_, *β*, *κ*-CN) and whey proteins (*β*-lactoglobulin, *α*-lactalbumin, lactoferrin, bovine serum albumin). Among these proteins, beta-casein (*β*CN) and beta-lactoglobulin (*β*LG) are of particular interest due to their significant differences in various aspects, including primary structure, secondary structure, tertiary structure, solubility, function, allergenicity, occurrence, and polymorphism. These differences distinguish *β*CN and *β*LG as distinct milk proteins with unique characteristics. In terms of primary structure, *β*CN consists of 209 amino acids, while *β*LG is composed of 162 amino acids, indicating a difference in their amino acid sequences. When it comes to secondary structure, *β*LG contains *α*-helical and *β*-sheet structures, whereas *β*CN is an intrinsically disordered protein and lacks regular secondary structures^2^. The differences extend to their tertiary structures as well. *β*-lactoglobulin has a well-defined tertiary structure with a hydrophobic ligand-binding pocket. In contrast, *β*-casein does not have a defined tertiary structure. The solubility of these proteins also varies. *β*-casein is highly soluble in water, while *β*-lactoglobulin is less soluble and can form aggregates when heated. Functionally, *β*CN plays a vital role in the stability of casein micelles and calcium binding, whereas *β*LG is a lipid and vitamin-binding protein. In terms of allergenicity, *β*LG is a major cow's milk allergen, while *β*CN is less allergenic. Furthermore, *β*-casein is specific to milk, whereas *β*-lactoglobulin also occurs in other body fluids^3^. Finally, both *β*-casein and *β*-lactoglobulin exhibit polymorphism, but the different forms of these proteins have different properties. For instance, different forms of *β*-casein (A1, A2, B) vary in their calcium-binding ability^4^, while different forms of *β*-lactoglobulin (A, B) differ in their thermal stability and allergenicity. In conclusion, *β*-casein and *β*-lactoglobulin, despite both being milk proteins, exhibit a wide range of differences in their structure, modifications, and properties.

Enzymatic hydrolysis of proteins leads to the release of fragments that can contribute to the overall health of various systems, including the immune, cardiovascular, nervous, and digestive systems^5^. Protein hydrolysates, primarily composed of dipeptides and tripeptides, are known to be absorbed more rapidly compared to free amino acids or intact proteins, thereby enhancing the absorption capacity of dietary supplements^6^. Furthermore, protein hydrolysates have found applications in animal nutrition^7^. Proteins frequently experience alterations in their native sequences through a process known as post-translational modifications (PTMs), which can profoundly impact their functional activity. One such PTM is phosphorylation. Post-translational modifications also vary between the two proteins. *β*-casein is, on one hand, phosphorylated at multiple serine residues, which is crucial for its calcium-binding ability^8^. On the other hand, *β*-lactoglobulin is not typically phosphorylated. Additionally, *β*-casein can be also glycosylated, which impacts its functional properties, while *β*-lactoglobulin is not typically glycosylated. However, the phosphorylation of *β*-lactoglobulin under artificial conditions has been observed in various studies^9^. In the study by Sitohy et al.^9^ different molar ratios of POCl_3_/protein were employed to phosphorylate *β*-lactoglobulin, in the presence of either triethylamine or hexylamine as the base. The use of aqueous conditions facilitated the phosphorylation process. Hence, the development of efficient and precise methods for studying protein digestion is of paramount importance.

A promising approach in the field of proteomic research is the use of microreactors with immobilized enzymes (μ-IMERs)^10^. These flow systems encapsulate or preserve the catalytic activity of enzymes, facilitating sequential or continuous digestion processes. The appeal of μ-IMERs over in-gel digestion lies in their numerous advantages, such as reduced digestion times, enhanced sample throughput due to improved mass transfer, increased enzyme concentration, and prevention of self-digestion. Additionally, the absence of enzyme molecules in the final sample and the extended reuse of μ-IMERs add to their appeal. Proteomics research predominantly employs μ-IMERs in conjunction with MS or MS/MS detection due to their capacity to provide valuable insights into peptide identification and potential modifications, such as oxidation or post-translational modifications^11^. Matrix-assisted laser desorption/ionization (MALDI) coupled with a time-of-flight (TOF) mass spectrometer is frequently used as the ion source in proteomic studies. Mass spectrometers serve as indispensable analytical tools for determining molecular mass, a critical factor for protein identification, characterization of chemical modifications, and structural analysis. Protein identification via MS techniques can be achieved through two primary methods: *bottom-up* and de novo sequencing analysis. The more commonly used *bottom-up* strategy involves the classical tryptic in-gel digestion or enzymatic digestion of proteins into peptides, which are then identified on the mass spectrometer. PTM analysis can largely be facilitated by PTM-specific protein and peptide enrichment methods, such as the use of ZipTips after the digestion step^12–14^. Subsequent MS and MS/MS analysis of peptides will increase sensitivity and specificity, reveal the identity of proteins, and allow specific assignment of modifications sites^15^. While numerous studies have focused on monolith-based enzyme microreactors for the detection and identification of digestion products using mass spectrometry, to our knowledge, no literature has reported a comparison between the classical in-gel digestion method and μ-IMER, in conjunction with the use of ZipTips for peptide enrichment^16,17^.

This study aimed to develop an efficient sample preparation method for identifying tryptic digest peptides of two model proteins found in cow's milk: *β*-casein (*β*CN) and *β*-lactoglobulin (*β*LG). These proteins were selected for their abundant nutrient content and functional attributes, notwithstanding their structural and compositional differences. We employed two protein digestion approaches: the traditional in-gel protein digestion method and enzymatic digestion using a flow-through microreactor (μ-IMER). Furthermore, we evaluated ZipTip pipette tips containing a chromatographic stationary phase for their impact on sequence coverage, peptide concentration, and purification. The activity of trypsin immobilized in the microreactor was assessed using the commercially available substrate BAEE, enabling the determination of optimal hydrolysis conditions. This holistic methodology enabled an in-depth analysis of peptides in *β*-casein and *β*-lactoglobulin, thereby enriching our knowledge of these essential bovine milk proteins.

## Materials and methods

### Materials

All chemicals used in this experiment were of analytical reagent grade or higher*. β*CN and *β*LG were purchased from Sigma-Aldrich (Steinheim, Germany). Deionized water was obtained from the Mili-Q ultrapure water producing system (Millipore, Bedford, MA, USA). Trypsin Gold, Mass Spectrometry Grade used for digestion in a solution were provided by Promega (Madison, Wisconsin, USA). Fused-silica capillaries (150 μm i.d. × 375 μm o.d.) were purchased from CM Scientific Ltd. (Dublin, Ireland); 3-(trimethoxysilyl)propyl methacrylate (*γ*-MAPS), glycidyl methacrylate (GMA), ethylene dimethacrylate (EDMA), azobisisobutyronitrile (AIBN), 1-dodecanol, cyclohexanol, sodium bicarbonate, benzamidine, sodium cyanoborohydride, the storage solution (containing sodium azide), N-*α*-benzoyl-l-arginine ethyl ester (BAEE), trypsin from bovine pancreas, trifluoroacetic acid (TFA), acetonitrile (HPLC ultra-gradient grade), ammonium bicarbonate, dichloromethane, methanol, sodium hydroxide were purchased from Sigma–Adrich (Steinheim, Germany); acetone and toluene were purchased from Chempur (Poland); 1,6-hexanediamine, glutaraldehyde, sodium phosphate monobasic dihydrate were purchased from Alchem (Poland). All the chemicals supplied for the MALDI-TOF MS analyses were at the highest commercially available purity by Fluka Feinchemikalien (Neu-Ulm, Germany; a subsidiary of Sigma–Aldrich).

### Instrumentation

Rheos 2000 HPLC pump (Flux Instruments, Reinach, Switzerland was used to flush the columns after monolith synthesis and for permeability measurements. A syringe pump NE-1002X (New Era Pump Systems, Farmingdale, NY) was used to pass reagents during the modification and trypsin immobilization and the substrate solution through the μ-IMER. The thermostat (Julabo, type F25) connected to a specially designed heat exchanger was used for reactions performed at a constant temperature. Chromatographic experiments were conducted in a nanoLC system consisting of a 1260 Capillary Pump (Agilent Technologies, Waldbronn, Germany), a 10-port nanoLC valve (model C72MX-6690D Vici-Valco, Schenkon, Switzerland) and a Crystal 100 UV–Vis detector (Thermo Separation Products, San Jose, CA, USA). The system was controlled using Clarity software (DataApex, Prague, Czech Republic).

MALDI-TOF MS mass spectra were acquired using a Bruker UltrafleX-treme II mass spectrometer operating at a repetition rate of 2 kHz in TOF mode and 1 kHz in TOF/TOF mode. A modified Nd:YAG laser emitting at a wavelength of 355 nm was utilized for the analyses. The experimental procedures and parameters followed in this study were in accordance with previously published protocols, ensuring consistency and comparability with prior work^18^.

### Classical in-gel protein digestion method with trypsin

#### Polyacrylamide gel electrophoresis study

To analyze the standard solutions of *β*CN and *β*LG, one-dimensional sodium dodecyl sulfate polyacrylamide gel electrophoresis (1D SDS-PAGE) was employed. The Bolt™ Mini Gel Tank from Novex Life Technologies (Carlsbad, CA, USA) was utilized for this purpose. SeeBlueTM Plus2 Pre-Stained Protein Standard, obtained from Novex Life Technologies in Europe (Bleiswijk, Netherlands) served as the molecular weight marker during the gel electrophoresis. Prior to 1D SDS-PAGE, proteins were purified using Amicon® Ultra centrifugal filter units (Merck, Poland).

#### Tryptic digestion in-gel

The trypsin digestion in-gel was performed following the established protocol described by Shevchenko et al.^19^. This protocol consisted of five key steps: (I) decolorization of the gel using 10 mM ammonium bicarbonate (NH_4_HCO_3_) with acetonitrile, (II) reduction of disulfide bonds using 10 mM dithiothreitol (DTT) in 10 mM NH_4_HCO_3_, (III) alkylation of thiols using 55 mM iodoacetamide (IAA) in 10 mM NH_4_HCO_3_, (IV) trypsin digestion carried out for 24 h, (V) extraction of peptides^20^.

To target phosphorylation and enrich modified peptides, ZipTips were employed. After the peptide extraction step, selected samples were subjected to ZipTips treatment, utilizing a chromatographic bed of 0.6 μL of C18 resin. This step aimed to concentrate and purify peptides containing modifications, thereby improving the sensitivity and resolution of MALDI-TOF MS analysis. Optimal binding to the ZipTip was achieved by incorporating 0.1% trifluoroacetic acid (TFA), which facilitated ion pairing.

### Capillary enzyme reactors (CER)

#### Conditions for preparation and modification of the capillary surface

First, the capillary (150 μm i.d. × 375 μm o.d.) was flushed with acetone, dichloromethane, and then dried by flushing with nitrogen. After that, the inner surface of the fused silica capillary was modified by etching with 1 M sodium hydroxide solution at 100 °C for 3 h followed by silanization with 10% *γ*-MAPS solution in toluene at 23 °C for 2 h.

#### Monolithic support synthesis

Polymerization solutions for the synthesis of a monolithic capillary column were prepared according to the procedure described in our previous work^18^. Namely, the polymerization solution consisted of a mixture of the functional monomer GMA and the crosslinking monomer EDMA in the presence of 1% initiator AIBN (relative to the weight of the monomers) and the porogen solution in a ratio of 40:60 (w/w). The weight ratio of the monomers was kept constant and equaled 3:2 (w/w), while the porogen solution consisted of 1-dodecanol and cyclohexanol in a ratio of 30:70 (w/w). In the next step, the polymerization mixtures were subjected to sonication, centrifugation, purging with nitrogen (to remove oxygen), and then injected into the capillaries using a syringe. The ends of the capillary were sealed with pieces of silicone rubber, after which the capillaries were placed in a water bath. The polymerization was carried out at 80 °C for 24 h.

The permeability of the produced monoliths (*K*_*F*_) was evaluated by measuring the flow rate of acetonitrile through the capillaries at a given pressure. The following formula was used:

1 $${K}_{F}=\frac{F\eta L}{\Delta P\pi {r}^{2}}\left[{m}^{2}\right]$$

where: *F*—flow rate (μl/min); $\eta$—acetonitrile viscosity (Pa s); *L*—the capillary length (dm), *ΔP*—pressure (Pa); *r*—the capillary radius (dm).

The obtained polymer was characterized by high permeability *K*_*F*_ = $4.85\times {10}^{-14} {\mathrm{m}}^{2}$ and homogeneous structure.

#### Trypsin immobilization

Immobilization of the enzyme was carried out in capillaries filled with monolithic GMA-co-EDMA support using a four-step method based on (I) initial aminolysis of the epoxide ring, (II) aldehyde attachment, (III) enzyme binding and (IV) final reduction of imine bonds. The first step was the aminolysis of epoxide rings performed using a 10% buffer of 1,6-diaminohexane at 80 °C for 2.5 h at a flow rate of F = 1 μl/min. The second step was the connection of glutaraldehyde dissolved in a buffer solution of pH 8.5 and at 23 °C for 3 h with a flow rate of 1 μl/min. Next, immobilization of the enzyme at a concentration of 3.5 mg/ml was carried out in the presence of 50 mM benzamidine at 4 °C for 24 h and a flow rate of F = 0.5 μl/min. The final step was the reduction of imine bonds using 0.1 M sodium cyanoborohydride solution (NaCNBH_3_) at 23 °C for 2 h at F = 1 μl/min. Solutions were circulated through the capillary bed using a syringe pump equipped with a system to regulate the temperature of both the capillary and the liquid being injected.

#### The efficiency of the μ-IMER produced

The efficiency of the obtained microreactor was evaluated by measuring the activity of trypsin using N-*α*-benzoyl-arginine ethyl ester (BAEE). To find the BAEE peak, standard solutions of the substrate at the concentration range of 0.5–75 mM were passed through the IMER at a syringe pump flow rate of F = 1 μl/min at 23 °C (room temperature) and 37 °C (physiological temperature). The IMER was connected on line to the injection loop of the nanoLC system, which allowed to inject the IMER effluent (containing the substrate and its digestion product) sequentially into the separation column. Separation was carried out using a nanoLC system equipped with a homemade column packed with the octadecyl stationary phase (HALO C18, 2.7 µm, Advanced Materials Technology, Wilmington, DE, USA). The mobile phase consisted of water and acetonitrile (both with 0.1% trifluoroacetic acid (TFA) at a flow rate of F = 5.0 μl/min. The chromatographic process was started with 20% acetonitrile (ACN) content in the mobile phase, and then the it was increased to a final 40% ACN value. Detection was performed at λ = 223 nm.

As the reaction product, N-*α*-benzoyl-l-arginine (BA) was separated from the substrate N-*α*-benzoyl-l-arginine ethyl ester (BAEE) on the C18 column, the degree of hydrolysis (%H) could be calculated from the peak area of the substrate from the difference in BAEE concentrations in solutions before and after the reaction on the μ-IMER. The calibration curve and the following formula was used:

2 $$\%H=\frac{{C}_{BAEE,st}-{C}_{BAEE}}{{C}_{BAEE,st}}\times 100\%$$

where: *C*_*BAEE,st*_—standard BAEE solution concentration before passing through a μ-IMER (mM), *C*_*BAEE*_—BAEE concentration in solution after passing the standard solution through a μ-IMER (mM).

#### Protein digestion in μ-IMER

*β*CN and *β*LG at a concentration of 45 pm/μl was dissolved in 50 mM ammonium bicarbonate buffer (ABC), pH 8.05. Using a syringe pump, proteins were passed through the μ-IMER at 37 °C and a flow rate of F = 0.05 μl/min. It corresponds to the residence time of 1653 s ≈ 28 min (capillary of 13.0 cm in length). The eluate was collected in a plastic vial and then analyzed with MALDI-TOF MS. Similar to the classic in-gel protein digestion method, some of the samples were subjected to an additional peptide enrichment step using ZipTips.

### Spectrometric study

Intact spectra were acquired using a positive ion linear mode, covering the m/z range of 5000–10,000. Sinapic acid (SA) served as the matrix for intact analyses, while Protein Calibration Standard II was employed for calibration purposes. On the other hand, for the peptide mass fingerprint (PMF) spectrum of trypsin-digested proteins, the positive reflectron mode was utilized, spanning the m/z range of 500–3500. In this case, *α*-cyano-4-hydroxycinnamic acid (HCCA) and 2,5-dihydroxybenzoic acid (DHB) were used as matrices, and Peptide Calibration Standard II served as the calibrant. A mass tolerance of 0.3 Da was set for all spectra, and internal calibration was performed on immonium ions. The measurements were conducted at an accelerating voltage of 25 kV. To apply the samples onto a MALDI steel target (AnchorChip), the dried drop method was employed.

**Figure 1:**
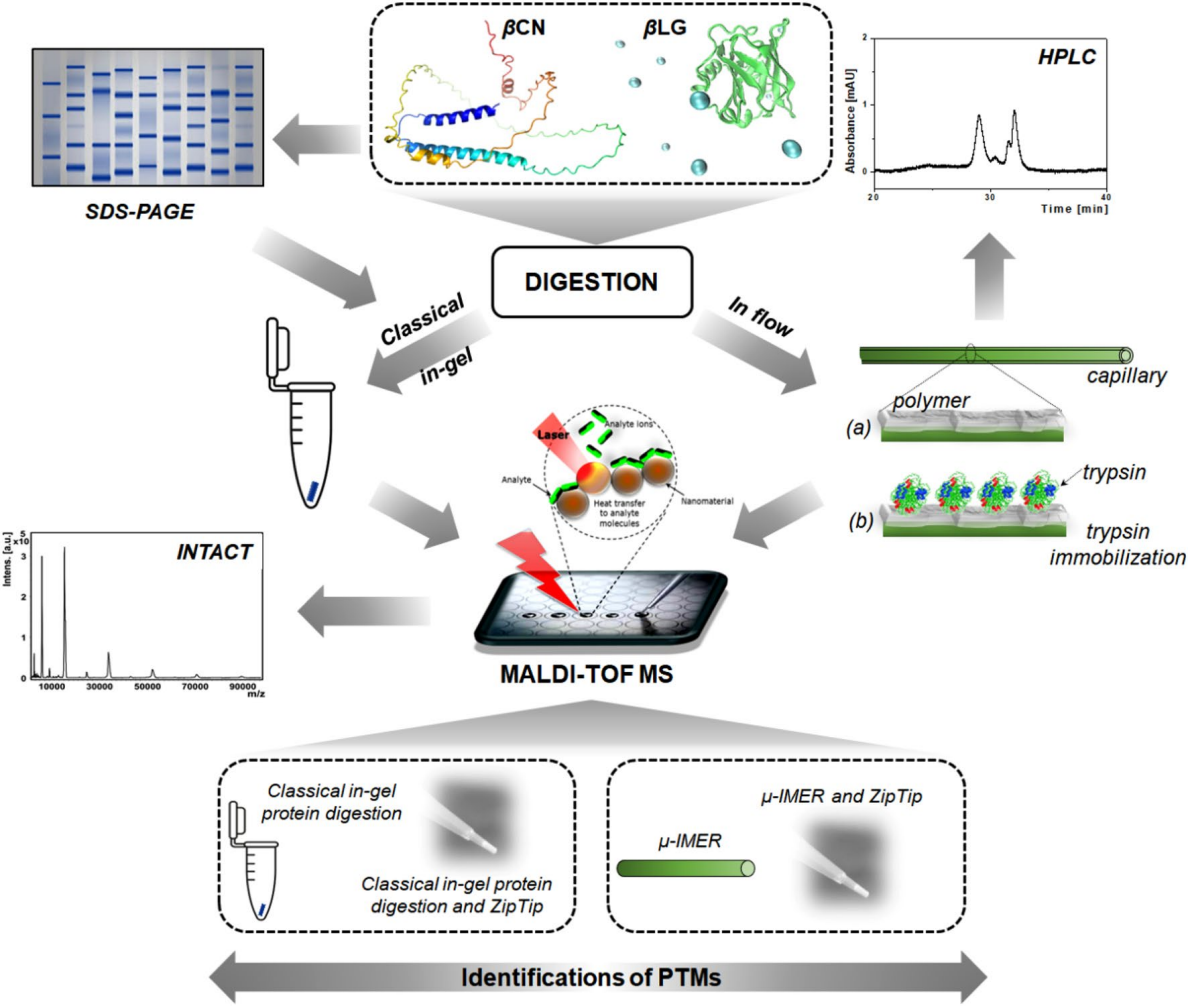
Schematic representation of the experimental design.

Software programs such as flexControl and flexAnalysis from Bruker Daltonik were utilized for acquiring and processing the mass spectra. Fragment spectra were collected using LIFT (Laser Ionization Fragmentation Technologies) in MS/MS mode to investigate both peptide sequences and their post-translational modifications, including phosphorylations, as well as oxidation. The resulting peptides generated from the trypsin digestion of *β*LG and *β*CN were identified using the BioTools software (Bruker Daltonik). The sequence coverage reported was based on the average of three replicate analyses. The experimental procedure followed the scheme illustrated in Fig. 1.

## Results and discussion

### Classical in-gel protein digestion method with trypsin

Gel electrophoresis is a widely used method for studying proteins, allowing for the separation of individual protein fractions based on differences in size, charge, or conformation. In the context of this study, gel electrophoresis was performed to analyze the proteins of interest, *β*CN and *β*LG. This technique provided information on the purity of the protein samples and offered a preliminary estimation of their molecular masses. Visual evaluation of the electrophoresis results (qualitative assessment) was conducted, as depicted in Fig. S1. The obtained bands indicated a mass of approximately 23 kDa for *β*CN and 18 kDa for *β*LG.

Following gel electrophoresis, protein bands were excised from the polyacrylamide gel, and an in-gel digestion process was carried out. This process included washing the gel pieces, followed by reduction and alkylation steps, in-gel digestion, and subsequent extraction of the resulting peptides, as described in the experimental section. Prior to spectrometric analysis, the peptide samples underwent purification and concentration using ZipTips. This step was performed to assess whether this particular sample preparation method could potentially improve the results obtained (Spectrometric study).

### Capillary enzyme reactors (CER): the efficiency of the μ-IMER produced

Attachment of the enzyme to the support structure is achieved by hydrophobic, electrostatic or covalent reaction interactions. Choosing the correct support can be a real challenge because the enzyme loading, porosity or pH stability must be taken into account. Many factors affect the effectiveness of digestion, these include the choice of support, residence time as well as the dynamics of the flow supporting the digestion. To allow enzyme–substrate interactions, a longer reaction (residence) time is usually required. The residence time needs to be increased if reduced efficiency is observed in IMERs in comparison to the free enzyme, or if reduced sequence coverage is observed by MS, particularly if no change in substrate affinity is observed^10^.

**Figure 2:**
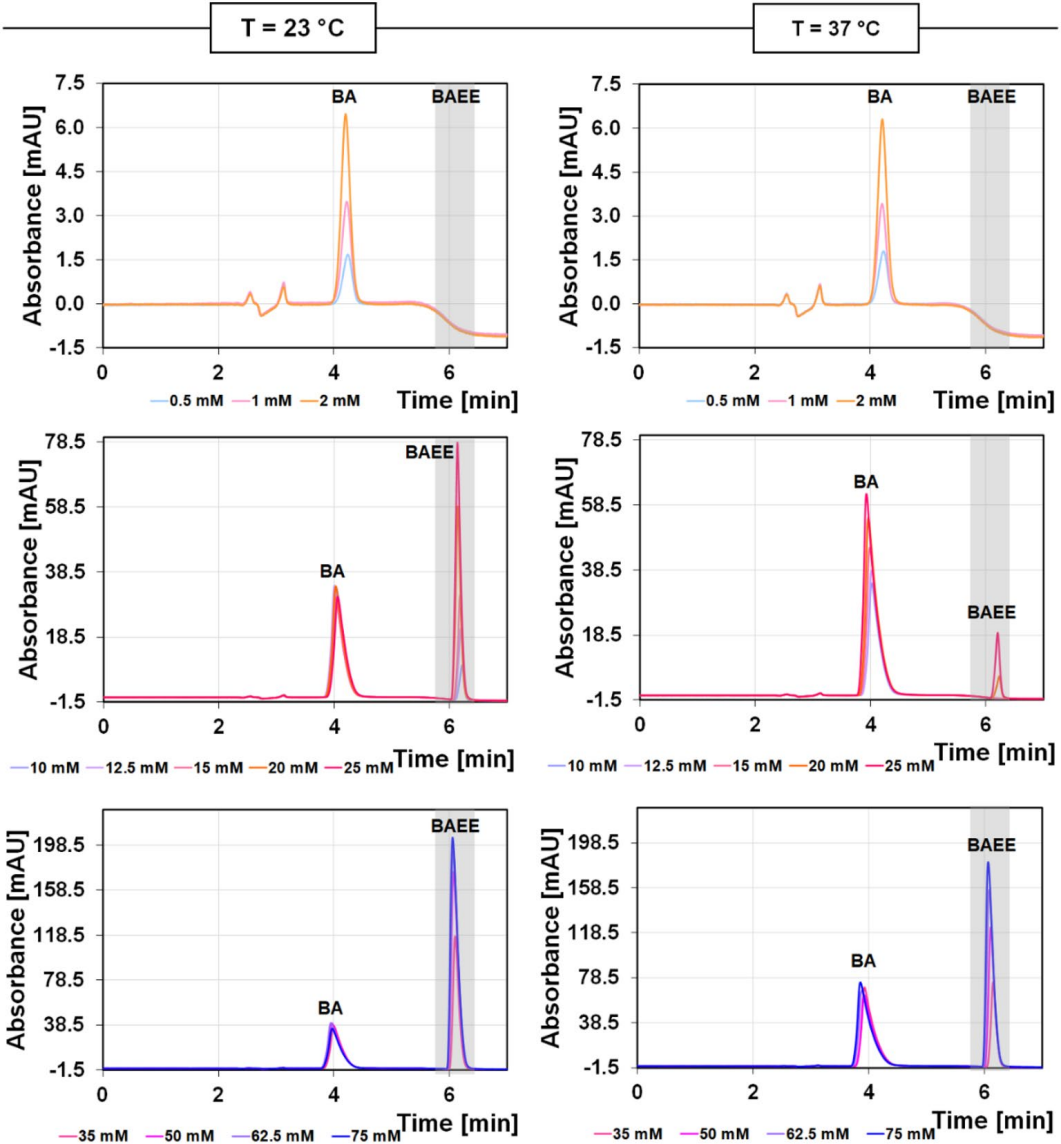
Effect of substrate concentration and temperature on the efficiency of the prepared microreactor at the flow rate of F = 1 μl/min.

The activity (efficiency) of the prepared microreactor was checked on the basis of the trypsin-catalyzed hydrolysis reaction of N-*α*-benzoyl-arginine ethyl ester (BAEE), as by the action of trypsin, BAEE is hydrolyzed to N-*α*-benzoyl-arginine (BA) and ethanol. Trypsin was chosen because it cleaves the C-terminal of arginine and lysine, thus leaving positively charged amino acids on the newly formed C-terminal, which promotes ionization and fragmentation^21^. For BAEE, the product (BA) of the reaction was separated from the substrate (BAEE) by capillary liquid chromatography, while the yield of the reaction was evaluated using the peak area of the substrate. Due to the similar absorption of radiation by both the substrate and the product, the detection was carried out at λ = 223 nm. Initially, isocratic elution with 40% acetonitrile (ACN) in the presence of 0.1% trifluoroacetic acid (TFA) was used, but due to the long analysis time, gradient elution was applied. The chromatographic process was carried out from 20% ACN content in the presence of TFA in the mobile phase, increasing it to 40%. TFA is the agent most commonly used to separate peptides and proteins in chromatography and acts as an ion-pairing agent. Figure 2 shows the effect of substrate concentration (0.5–75 mM) and temperature (23 °C and 37 °C) on the efficiency of the prepared microreactor at the applied flow rate F = 1 μl/min.

In our study, we explored the hydrolysis efficiency of BAEE using μ-IMER under different conditions. For solutions with low concentrations of BAEE (0.5 mM, 1 mM, and 2 mM) at room temperature (23 °C), we observed complete hydrolysis, as evidenced by the absence of substrate signals in the eluate. This suggests that the μ-IMER is highly effective at these concentrations. However, for higher concentrations ranging from 10 to 75 mM, the degree of hydrolysis varied between 91.23 and 27.74%. This incomplete hydrolysis is evidenced by the presence of unreacted ester in the eluate, and could be attributed to the saturation of enzymatic sites, hence—insufficient contact time between the enzyme and substrate molecules. So, this problem can be overcome (at least partially) by increasing the enzymatic activity (by providing optimum temperature conditions) and by lowering the flow rate to increase contact time between trypsin and digested molecules. It is also worth noting here that BAEE can undergo spontaneous hydrolysis. According to the literature data, approximately 4% of BAEE is spontaneously hydrolyzed within 24 h when left at room temperature, while the solution remains stable for at least 14 days when stored at 4 °C^22^. Therefore, the observed hydrolysis in our study is likely enzymatic rather than spontaneous, especially at lower concentrations. When the temperature was increased to 37 ºC, which is closer to the optimal temperature for trypsin activity, complete hydrolysis was again observed for low concentrations (0.5 mM, 1 mM, and 2 mM) as well as for some higher concentrations (10 mM, 12.5 mM, and 15 mM)^23^. For concentrations ranging from 20 to 75 mM, the degree of hydrolysis was between 98.60 and 36.24%. Given that 37 °C is the optimal temperature for trypsin, we chose this temperature for further studies involving proteins *β*CN and *β*LG. This comprehensive analysis underscores the importance of optimizing both substrate concentration and reaction temperature to achieve efficient hydrolysis, particularly when using μ-IMERs for complex biological samples^23^.

### Capillary enzyme reactors (CER): protein digestion in μ-IMER

The use of liquid chromatography allowed the separation of milk proteins *β*CN and *β*LG. Considering that protein digestion requires longer contact time with trypsin compared to the model BAEE ester, a lower flow rate of F = 0.05 μl/min was used.

**Figure 3:**
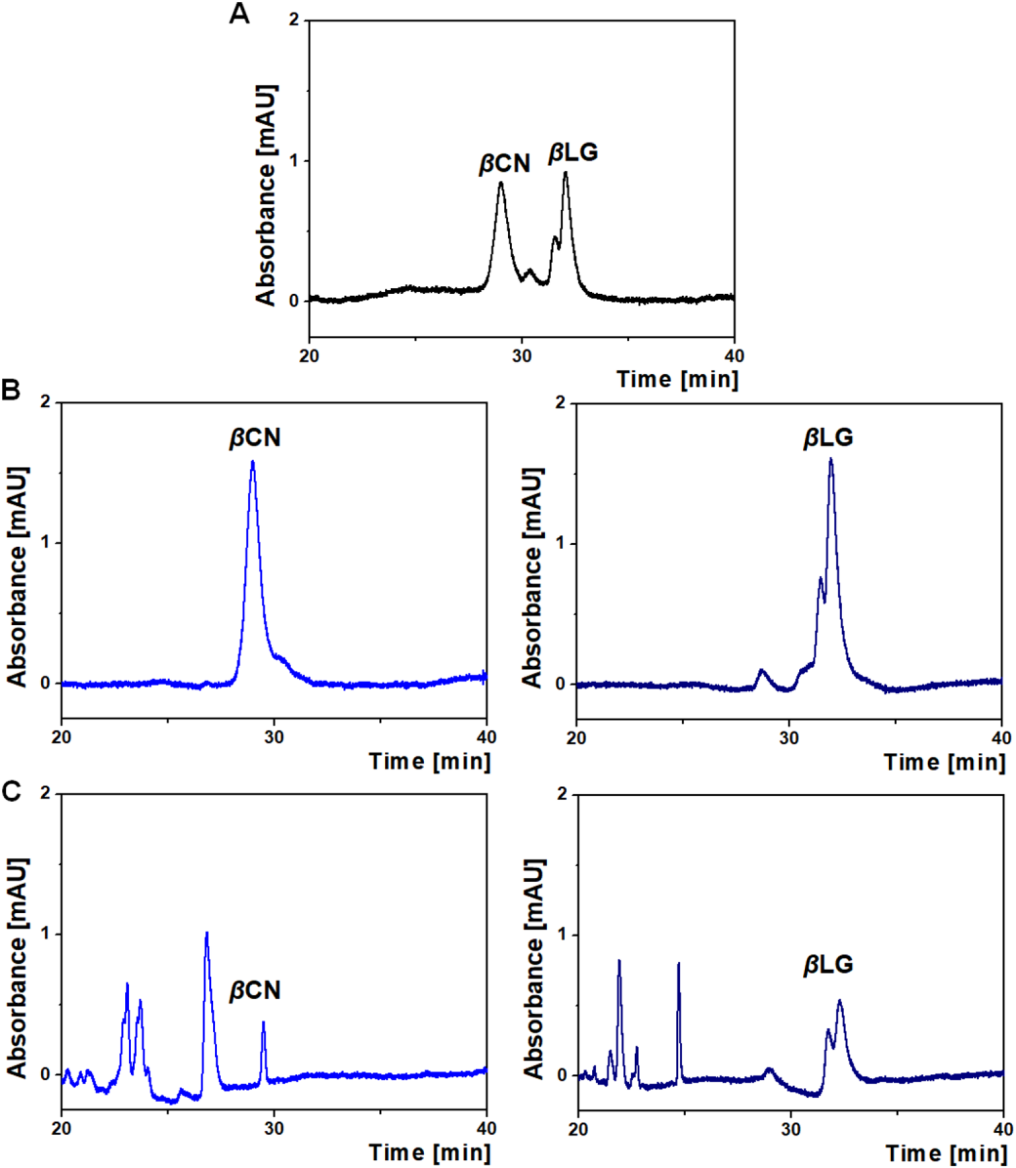
Chromatographic profile of a mixture of *β*CN and *β*LG (**A**), native proteins (**B**), and proteins passed through a microreactor (**C**).

The chromatograms (Fig. 3) show very good quality separation and clear signals from the studied proteins.

The first eluted protein in the mixture was *β*CN, then *β*LG. The retention times of the eluted analytes were consistent with those observed for *β*CN and *β*LG standards. In addition, two signals were observed for *β*LG, which are derived from its genetic variants. A quick and easy way to prepare the sample for the HPLC separation was developed. Proteins that passed through a microreactor were subjected to analogous tests. It was observed that the intensity of signals for both *β*CN and *β*LG decreased compared to the native form of both proteins and additionally new signals appeared. The results indicate the activity of the microreactor towards the proteins studied.

### Spectrometric study

**Figure 4:**
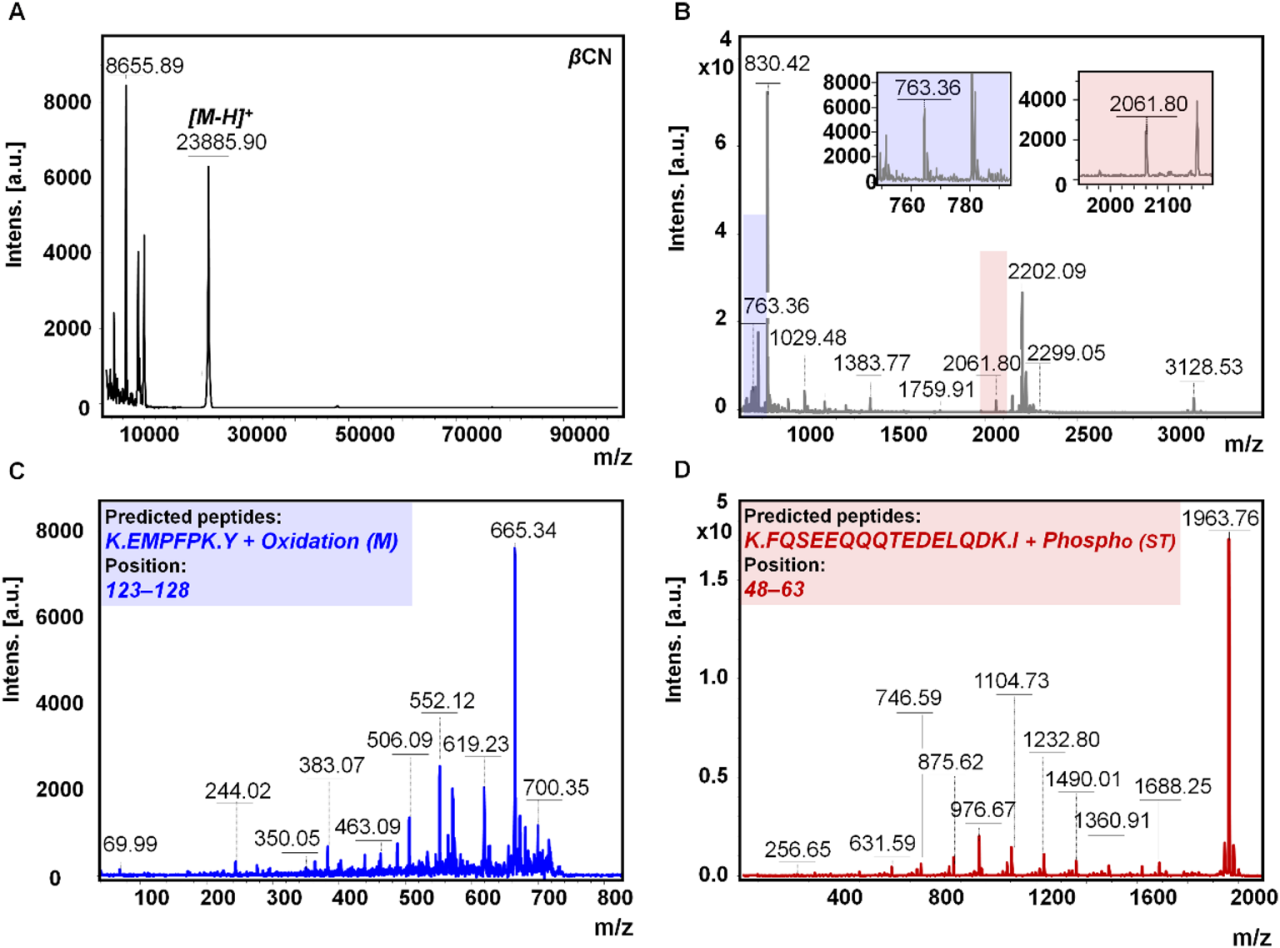
Schematic diagram illustrating the distinct signal patterns of *β*CN as captured in MALDI MS analysis. The mass spectra in MALDI-TOF MS mode display the components of *β*CN in their intact state (**A**); the spectrum obtained following trypsin digestion of *β*CN (**B**); the fragmentation of the signal at m/z = 763.36 (**C**); and the fragmentation of the signal at m/z = 2061.80 (**D**), all acquired using LIFT-TOF/TOF MS mode.

**Figure 5:**
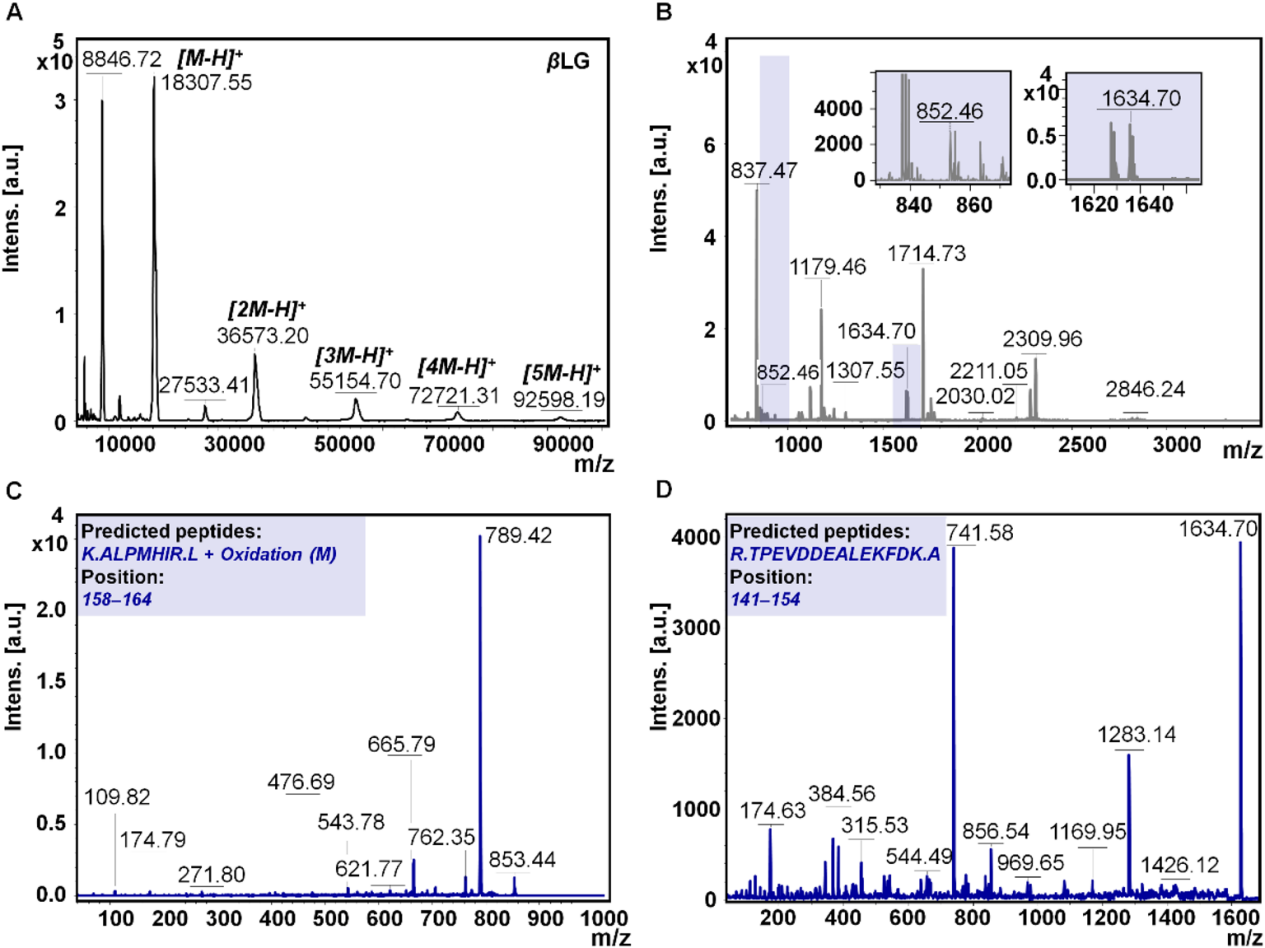
Schematic diagram illustrating the distinct signal patterns of *β*LG as observed in MALDI MS analysis. The mass spectra in MALDI-TOF MS mode show the components of *β*LG in their intact state (**A**); the tryptic digestion spectrum recorded for *β*LG (**B**); the fragmentation of the signal at m/z = 852.46 (**C**); and the fragmentation of the signal at m/z = 1634.70 (**D**), all captured using the LIFT-TOF/TOF MS mode.

Proteomic research heavily relies on mass spectrometry (MS) coupled with various sample preparation techniques and separation methods. However, the complexity of proteomic samples poses significant challenges in developing fast and efficient sample preparation methods for subsequent MS analysis^24^. In our study, we conducted a detailed characterization of the primary structure of two proteins, *β*CN and *β*LG. Through careful analysis, we determined the molecular weights of intact *β*CN (Fig. 4A) and *β*LG (Fig. 5A) to be approximately 23,885.90 ± 0.216 Da and 18,307.55 ± 0.145 Da, respectively. Additionally, besides the monomeric forms of the proteins, the presence of dimers, trimers, and in the case of *β*LG, even tetramers and pentamers, was observed. Interestingly, in the case of *β*LG, we also observed the presence of dimers [2M–H]^+^ and various oligomers such as trimers [3M–H]^+^, tetramers [4M–H]^+^, and pentamers [5M–H]^+^ (Fig. 5A). These findings align with the information provided in our previous research^3^ provides valuable insights into oligomeric forms study of *β*LG.

To gain further insights, the proteins underwent extensive analysis focusing on modifications to their peptides. Tryptic digestion was performed, and the resulting peptides were subjected to PMF analysis in a positive mode (Figs. 4B, 5B). Peptides were identified using BioTools software, employing non-standard cysteine searches modified to account for carbamidomethylation, oxidation, and phosphorylation. Tables 1S and 2S present the individual masses of the detected peptides, their sequences, the sites of oxidation and phosphorylation, and the sequence coverage considering above mentioned two different sample preparation methods: with and without the use of ZipTips. The phosphorylation was observed specifically on serine residues. The mass spectra of the identified protein peptides are shown in Figs. 4C,D and 5C,D.

The in-depth analysis provided valuable information regarding the peptide sequences, and their modifications, contributing to a comprehensive understanding of the proteins *β*CN and *β*LG in this study.

**Figure 6:**
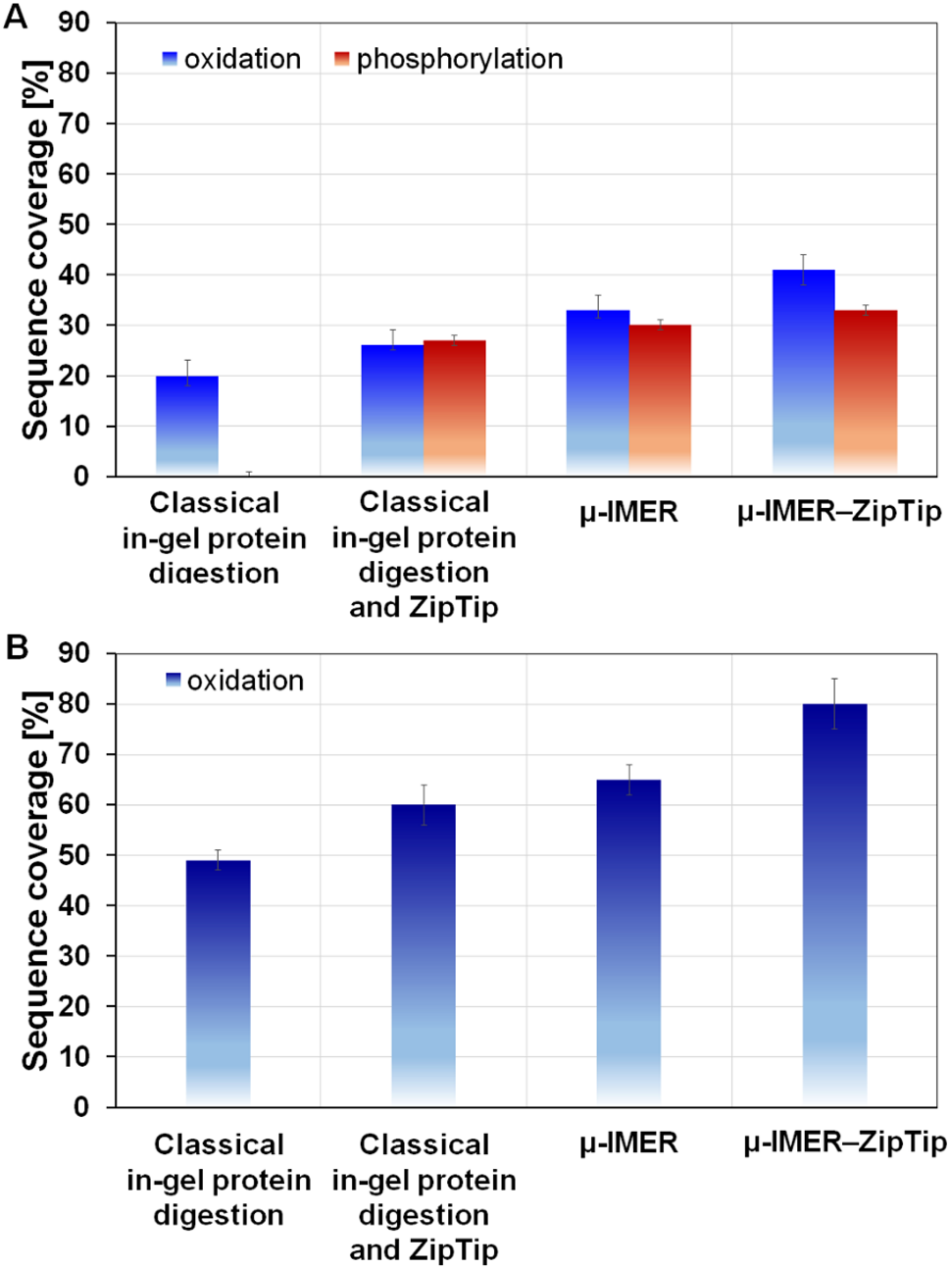
Sequence coverage using different sample preparation methods for analysis to *β*CN (**A**) and *β*LG (**B**).

To identify the proteins *β*CN and *β*LG, enzymatic digestion was performed using two different methods: the classical in-gel protein digestion method and the μ-IMER method. The aim was to determine the sample preparation method that would provide the highest degree of sequence coverage, allowing for more accurate protein identification (Fig. 6). In the classical method, the proteins were digested in-gel following the established protocol. For the μ-IMER method, the proteins were subjected to digestion in the flow-through microreactor, which offers advantages such as shorter digestion times and improved mass transfer. Additionally, to assess the impact of ZipTips on sequence coverage, some samples underwent peptide enrichment using this technique. By comparing the results obtained from the different digestion methods and sample preparation techniques, the most optimal approach for achieving the highest sequence coverage was determined. This selection is crucial for accurate protein identification, enabling a comprehensive understanding of the *β*CN and *β*LG proteins. The evaluation of the various methods and techniques allowed for the identification of the most suitable sample preparation method for subsequent analyses.

The findings reveal that the sequence coverage for the protein *β*CN, when subjected to classical in-gel protein digestion was 20 ± 2%, indicating that a fifth of the protein's sequence was analyzed and identified. This is specifically referring to the presence of oxidized molecules in the protein's structure. However, when the ZipTips were used in conjunction with the classical digestion, there was an improvement in the sequence coverage by 6%, increasing it to 26 ± 1%. These ZipTips, therefore, appear to enhance the detection and analysis capabilities of the sequence coverage process. The use of a flow-through microreactor, further boosted the sequence coverage, bringing it to 33 ± 1.5%. This shows that the μ-IMER provides a more efficient environment for protein digestion, leading to an increased understanding of the protein's sequence. This value was further enhanced to 41 ± 3%, when the ZipTips were applied after IMER digestion, meaning the combination of the μ-IMER and ZipTip was particularly effective in improving sequence coverage. This method, in fact, provided double the sequence coverage compared to the classical digestion method alone. In the case of *β*-lactoglobulin the classical digestion method offered significantly higher sequence coverage—49 ± 2% without ZipTip and 60 ± 4% with ZipTip. Again, using the μ-IMER led to an increase in sequence coverage up to 65 ± 3%, while with IMER-ZipTip combination sequence coverage reached 80 ± 5%, which is notably higher than the results achieved with the *β*CN protein. In our study, we utilized laser ionization fragmentation technique in MS/MS mode (LIFT TOF/TOF MS) to explore phosphorylations. Metastable fragmentation of the selected ions was achieved using a laser ionization, eliminating the need for additional collision gas^25^. Interestingly, no phosphorylation was detected in *β*CN when analyzed directly. However, the incorporation of ZipTips after classical digestion led to a marked increase in the sequence coverage for phosphorylated *β*CN sequences, reaching 27 ± 4%. This coverage was further enhanced to 30 ± 2% and 33 ± 1% after using μ-IMER and μ-IMER-ZipTip, respectively. For *β*LG, the use of ZipTip enabled the detection of a significantly higher proportion of modified peptides in both classical digestion (79 ± 2%) and μ-IMER (79 ± 4%). These findings underscore the utility of ZipTip in enhancing the detection of oxidized molecules and phosphorylations, particularly in the context of *β*CN, with similar improvements observed for *β*LG. The data suggest that the combined use of ZipTip and μ-IMER offers a more effective strategy for achieving comprehensive protein sequence coverage and post-translational modification analysis. Additionally, our μ-IMER method revealed the presence of new peptides that were not identified using the classical method, such as those with m/z values of approximately 763 and 3109 for *β*CN, and approximately 2706, 2722, and 2312 for *β*LG. Similarly, the use of ZipTip led to the identification of new peptides when compared to both the classical method and μ-IMER, with m/z values of approximately 645, 763, and 871 for *β*CN, and approximately 902 and 2312 for *β*LG in the classical method, and approximately 2235 for *β*CN and approximately 902 and 1248 for *β*LG in μ-IMER.

The concept of utilizing ZipTip is akin to the traditional solid-phase extraction (SPE) technique. The primary purpose of these methods is to augment the concentration of the analytes (components of interest) within the sample and, if required, to purify the sample by eliminating unwanted substances. Both these processes can enhance sequence coverage, which is achieved by amplifying the number of analyte molecules that undergo the MALDI process. Additionally, the removal of potential contaminants contributes to a more accurate and unobstructed analysis^26–28^. However, it's important to note that while these processes increase the concentration of the analytes, they might also inadvertently preconcentrate unwanted impurities^27^. Hence, one should remain cautious about this possibility during such operations. In the study under discussion, it's clear that the application of ZipTip amplified the degree of sequence coverage in the case of oxidized *β*CN and *β*LG, and also enhanced the detection of phosphorylated *β*CN in both the classical in-gel protein digestion and μ-IMER methods. Nevertheless, it is observable that the sequence coverage was superior when the μ-IMER method was used compared to the classical in-gel protein digestion. In particular, the use of the combined μ-IMER-ZipTips methodology yielded the best results for *β*CN. These results emphasize the μ-IMER's potential for preparing samples swiftly for MALDI-TOF MS analysis. As such, it is an encouraging prospect for advancing the field of proteomics, which involves the large-scale study of proteins, their structures, and functions. By enhancing the sequence coverage and improving the detection of post-translational modifications like oxidation and phosphorylation, these methods could significantly improve the quality and depth of proteomic studies.

A key type of PTM is phosphorylation, which is essentially the addition of a phosphate group to a protein. Phosphorylations have an instrumental role in a wide range of cellular activities^29,30^. For instance, abnormalities in phosphorylation processes are associated with the development of cancer and neurodegenerative disorders^31–33^. This makes the analysis of phosphorylation and other PTMs an essential component of disease research and potential therapeutic intervention strategies. However, the analysis of PTMs presents several challenges, particularly in the context of MS and MS/MS, which are widely used analytical techniques in proteomics^21,34,35^. Some PTMs are unstable during these analyses and may break down or change, making their identification and characterization difficult. Furthermore, several modifications result in the formation of hydrophilic (water-attracting) products. These products can make the handling and purification of PTM samples prior to MS analysis more complex, as they may not interact favorably with the commonly used techniques that are designed for hydrophobic (water-repelling) molecules^15^. PTMs can also affect the efficiency of proteases, such as trypsin, which are enzymes used to break down proteins into smaller peptides for easier analysis. PTMs can lead to unusual cleavage patterns or larger-than-expected peptide products, complicating the interpretation of results^15^. Additionally, the presence of certain PTMs can reduce the ionization and detection efficiency in MS. Ionization is a crucial step in MS where molecules are charged so they can be moved and measured by an electric or magnetic field. Reduced ionization can lead to lower detection levels, potentially obscuring significant findings. Lastly, when a protein has multiple PTMs, the resulting MS and MS/MS data sets can be incredibly complex and difficult to interpret. Each modification can affect the protein's behavior in the MS, leading to a wide array of potential products and signals to be interpreted^15^. Overall, while the analysis of PTMs can offer critical insights into protein function and disease development, it also presents various challenges that require sophisticated techniques and careful interpretation of the generated data.

There is another issue that make IMER based methodologies particularly effective. It is known, that in proteomic analyses, enzyme autolysis is a significant factor that can introduce complexity and potential inaccuracies in identifying and quantifying target peptides. Traditional in-gel digestion methods, while generally effective, often yield a complex polypeptide mixture. This mixture comprises fragments from both the target protein and the protease, complicating subsequent analyses. In our study, we observed trypsin autolysis signals in the in-gel digestion of *β*CN at specific m/z values (~ 842 and ~ 1045) with corresponding intensities of 3682 and 1375. Additional signals were also noted at m/z values of 3092 and 2299, albeit with much lower intensities (~ 350). These findings align with existing literature that discusses the implications of trypsin autolysis in in-gel digestion methods^36^. In the case of *β*LG, we detected multiple autolysis signals at various m/z values (732; 842; 870; 1045; 1178), but these signals exhibited low intensities (ranging from 300 to 900). This suggests that the impact of autolysis may be less significant for *β*LG under our experimental conditions. Importantly, we utilized a specialized form of trypsin in which lysine residues have been modified by a reductive methylation. This modification yields a trypsin molecule that is both highly active and extremely resistant to autolytic digestion, thereby potentially mitigating the issue of autolysis. Remarkably, our μ-IMER method showed no evidence of trypsin autolysis. This absence could be attributed to the enzyme's immobilization on the microreactor, which may confer stability and reduce its susceptibility to autolysis^36^. This finding not only highlights the utility of μ-IMER in proteomic analyses but also warrants further studies to explore the stability and reusability of immobilized trypsin in such systems.

This additional layer of complexity complicates the composition of the polypeptide mixture and makes subsequent analysis of the MS spectra more challenging. Autolysis peaks can suppress peptide signals in the MS spectra, thereby reducing the sensitivity of the method. To address these issues, one strategy is the immobilization of enzymes on solid supports or other carriers. By fixing the enzymes onto a solid structure, autolysis can be minimized while maintaining a high enzyme-to-substrate ratio, which is crucial for efficient protein digestion^37^. Such an approach offers several notable advantages over traditional in-gel protein digestion. First, it greatly accelerates the digestion process, enabling higher throughput than common protocols used in classical methods, such as those involving gel preparation. Furthermore, the μ-IMER method reduces the complications associated with sample handling and processing. For example, it can minimize concerns related to pipette transfer, which is a common source of errors and variability in traditional laboratory protocols^38^. By streamlining and improving the digestion process, the μ-IMER method can enhance the reliability and efficiency of proteomic analyses. The majority of the literature focuses on off-line microreactors, which although easier to implement, do not offer the real-time analysis capabilities of on-line systems^38^. The challenge in achieving on-line coupling, particularly with MS, lies in the difficulty of harmonizing the experimental conditions across different units in the workflow. This is a significant hurdle that our work aims to address. While some studies have managed to achieve on-line coupling through liquid chromatography (LC) or capillary electrophoresis (CE)^38^, our system is designed to be more versatile and robust, capable of on-line coupling with MS. In terms of efficiency and time, the study by Villegas et al. demonstrated that on-line digestion, separation, and characterization of proteins could be achieved in less than 30 min using commercial cellulose resin particles with immobilized trypsin^38^. Our system, while also aiming for high efficiency, has a unique focus on robustness and longevity. The unique monolithic support and capillary surface modifications contribute to its extended lifespan, making it a cost-effective option. Another study showed that an efficient IMER could significantly reduce the analysis time by approximately 15 h compared to classical solution digestion^39^. Our IMER not only aims to shorten the analysis time but also provides comparable or superior protein sequence coverage.

Different variants of protein modifications, including post-translational modifications, may influence their overall behavior and properties which might be particularly valuable for the development of functional foods and dietary supplements, such as protein hydrolysates. In this context, the methodology introduced in this study seems to be considerably promising in functional food analysis. The enhanced sequence coverage achieved through the use of μ-IMER and ZipTip, especially for proteins with complex PTMs, underscores the potential of these techniques to make significant contributions to protein research. Additionally, the speed and accuracy offered by these methods make them highly applicable in various fields that require rapid and precise protein analysis, including the development of functional foods and dietary supplements.

## Conclusions

This study offers a comprehensive comparison of proteomic methodologies, focusing on classical in-gel protein digestion, the use of a flow-through enzymatic microreactor (μ-IMER), and the application of ZipTip pipette tips. All methods were followed by MALDI-TOF MS for analysis. We centered our research on two bovine milk proteins, *β*-casein and *β*-lactoglobulin, which are characterized by their unique structural and molecular weight attributes. Our findings underscore the distinct advantages of using μ-IMER, which yielded superior sequence coverage of 33 ± 1.5% for *β*CN and 65 ± 3% for *β*LG, thereby promoting a more streamlined digestion process compared to classical in-gel digestion, which yielded 20 ± 2% for *β*CN and 49 ± 2% for *β*LG. The incorporation of ZipTip into both methods significantly enhanced sequence coverage, reaching 26 ± 1% for *β*CN and 60 ± 4% for *β*LG in classical in-gel digestion, and 41 ± 3% for *β*CN and 80 ± 5% for *β*LG in μ-IMER. It is important to note that sequence coverage was sensitive to variations in protein structure. Specifically, *β*LG, the smaller of the two proteins, demonstrated higher sequence coverage results. For oxidized *β*LG, sequence coverage increased steadily, peaking when both μ-IMER and ZipTip techniques were employed. In the case of *β*CN, the use of ZipTip in classical digestion enhanced sequence coverage to 27 ± 4%, and further increased to 30 ± 2% and 33 ± 1% when μ-IMER and μ-IMER–ZipTip were used, respectively. Additionally, ZipTip allowed for the detection of a higher percentage of modified peptides in both classical digestion (79 ± 2%) and μ-IMER (79 ± 4%) for *β*LG.

Our study suggests an innovative pathway to protein analysis by amalgamating classical in-gel digestion, μ-IMER, and ZipTip techniques. It sheds light on the pros and cons of these methods when dealing with diverse proteins, underscoring the impactful role of protein structure in the final outcomes.

Looking ahead, it would be worthwhile to test these methodologies on a wider variety of proteins to gain further insights into the impact of different protein structures on sequence coverage and overall analytical results. Exploring the use of additional proteases or combinations thereof could potentially enhance digestion efficiency. Through ongoing research and development, we can aspire to broaden and deepen our understanding of protein biology and its manifold applications.

### Supplementary Information

Supplementary Information.

## Data Availability

All data generated or analysed during this study are included in this published article [and its Supplementary information files].
