# Supplementary material for: Immobilized enzyme microreactors for analysis of tryptic peptides in β-casein and β-lactoglobulin
Source: Sci Rep. 2023 Oct 2;13:16551. doi: 10.1038/s41598-023-43521-z (PMC10545664; doi:10.1038/s41598-023-43521-z)
Supplement: Supplementary file 1 — Supplementary Information. [file 41598_2023_43521_MOESM1_ESM.pdf]

Immobilized Enzyme Microreactors for Analysis of Tryptic Peptides in *β*-Casein and *β*-Lactoglobulin

Agnieszka Rodzik[a,b], Viorica Railean[a,c], Paweł Pomastowski[a], Bogusław Buszewski[a,b], Michał Szumski[a]

[a] *Centre for Modern Interdisciplinary Technologies, Nicolaus Copernicus University in Toruń, Wileńska 4, 87-100, Toruń, Poland*

[b] *Department of Environmental Chemistry and Bioanalysis, Faculty of Chemistry, Nicolaus Copernicus University in Toruń, Gagarina 7, 87-100 Toruń, Poland*

[c] *Department of Infectious, Invasive Diseases and Veterinary Administration, Institute of Veterinary Medicine, Nicolaus Copernicus University in Torun, Gagarina 7, 87-100 Toruń, Poland*

**Corresponding author:* Agnieszka Rodzik (agnieszka.rodzik94@gmail.com), Michał Szumski (michu@umk.pl)

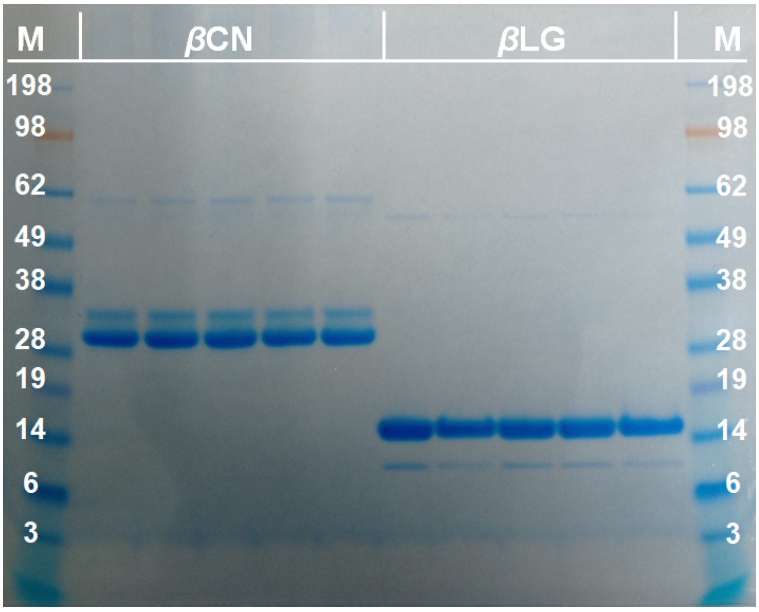

Fig. S1. SDS-PAGE electropherogram for *β*CN and *β*LG.

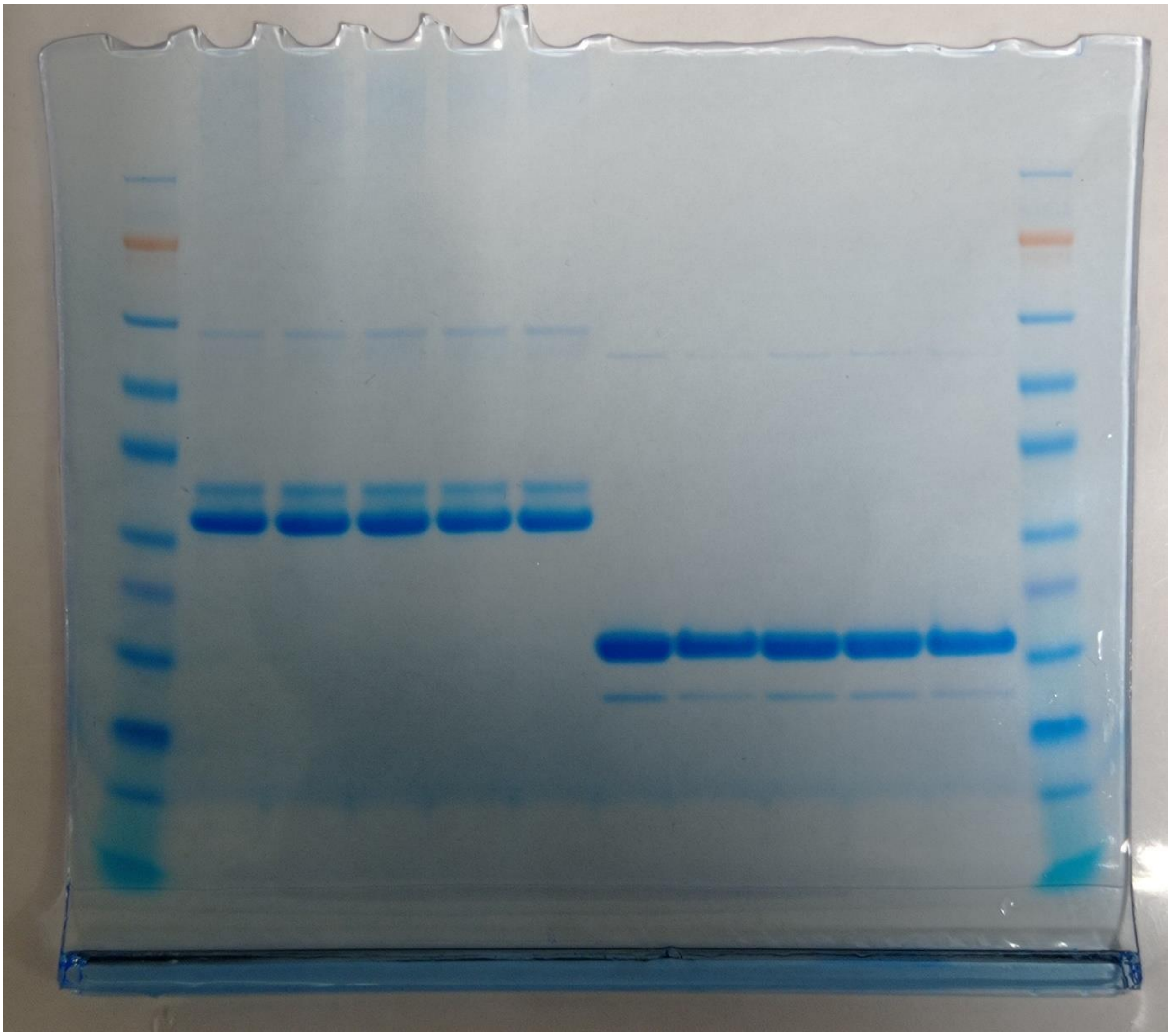

Fig. S1a. Original image of SDS-PAGE electropherogram for βCN and βLG in a non-reduced mode.

Table S1
The individual masses of detected peptides, their sequences, and the degree of sequence coverage considering the two methods of sample preparation with and without ZipTip pipette tips for βCN.

Sample preparation	Mass [Da]		Sequence coverage [%]	Sequence range	Sequence
	Theoretical	Measured			
**A** Classical in-gel protein digestion	747.38	747.36	20	123–128	K.EMPFPK.Y
	779.50	779.49		185–191	K.VLPVPQK.A
	829.47	829.44		192–198	K.AVPYPQR.D
	2185.13	2185.16		199–217	R.DMPIQAFLLYQEPVLGPVR.G
	2201.08	2201.16		199–217	R.DMPIQAFLLYQEPVLGPVR.G + Oxidation (M)
	741.44	741.44		218–224	R.GPFPIIV.-
**B**	871.45	871.55	26	41–47	R.INKKIEK.F
	645.18	645.32		115–120	K.EAMAPK.H
	747.32	747.36		123–128	K.EMPFPK.Y

Classical in-gel protein digestion and ZipTip	763.36	763.36		123–128	K.EMPFPK.Y + Oxidation (M)
	779.47	779.49		185–191	K.VLPVPQK.A
	829.43	829.44		192–198	K.AVPYPQR.D
	2185.22	2185.16		199–217	R.DMPIQAFLLYQEPVLGPVR.G
	2201.21	2201.16		199–217	R.DMPIQAFLLYQEPVLGPVR.G + Oxidation (M)
	741.45	741.44		218–224	R.GPFPIIV.-
	2061.80	2060.82	27	48–63	K.FQSEEQQQTEDELQDK.I + Phospho (ST)
	748.39	747.36		123–128	K.EMPFPK.Y
	780.51	779.49		185–191	K.VLPVPQK.A
	830.48	829.44		192–198	K.AVPYPQR.D
	2186.14	2185.16		199–217	R.DMPIQAFLLYQEPVLGPVR.G
	742.45	741.44		218–224	R.GPFPIIV.-
**C** μ-IMER	3109.41	3109.57	33	17–44	R.ELEELNVPGEIVESLSSSEESITHINKK.I
	747.33	747.36		123–128	K.EMPFPK.Y
	763.38	763.36		123–128	K.EMPFPK.Y + Oxidation (M)
	779.46	779.49		185–191	K.VLPVPQK.A
	829.42	829.44		192–198	K.AVPYPQR.D
	2185.04	2185.16		199–217	R.DMPIQAFLLYQEPVLGPVR.G
	2201.02	2201.16		199–217	R.DMPIQAFLLYQEPVLGPVR.G + Oxidation (M)
	741.41	741.44		218–224	R.GPFPIIV.-
	871.41	871.55	30	41–47	R.INKKIEK.F
	2060.69	2060.82		48–63	K.FQSEEQQQTEDELQDK.I + Phospho (ST)
	747.33	747.36		123–128	K.EMPFPK.Y
	779.46	779.49		185–191	K.VLPVPQK.A
	829.41	829.44		192–198	K.AVPYPQR.D
	2185.04	2185.16		199–217	R.DMPIQAFLLYQEPVLGPVR.G
	741.41	741.44		218–224	R.GPFPIIV.-
**D** μ-IMER and ZipTip	3109.77	3109.57	41	17–44	R.ELEELNVPGEIVESLSSSEESITHINKK.I
	2235.99	2236.20		64–83	K.IHPFAQTQSLVYPFPGPIPK.S
	747.20	747.36		123–128	K.EMPFPK.Y
	763.29	763.36		123–128	K.EMPFPK.Y + Oxidation (M)
	779.28	779.49		185–191	K.VLPVPQK.A
	829.33	829.44		192–198	K.AVPYPQR.D
	2185.11	2185.16		199–217	R.DMPIQAFLLYQEPVLGPVR.G
	2201.12	2201.16		199–217	R.DMPIQAFLLYQEPVLGPVR.G + Oxidation (M)
	741.32	741.44		218–224	R.GPFPIIV.-
	2060.80	2060.82	33	48–63	K.FQSEEQQQTEDELQDK.I + Phospho (ST)
	747.40	747.36		123–128	K.EMPFPK.Y

2262.09	2262.03		129–147	K.YPVEPFTESQSLTLTDVEK.L + Phospho (ST)
779.53	779.49		185–191	K.VLPVPQK.A
2185.08	2185.16		197–215	R.DMPIQAFLLYQEPVLGPVR.G
2185.32	2185.16		197–215	R.DMPIQAFLLYQEPVLGPVR.G
741.49	741.44		216–222	R.GPFPILV.-

Table S2
The individual masses of detected peptides, their sequences, and the degree of sequence coverage considering the two methods of sample preparation with and without ZipTip pipette tips for βLG.

Sample preparation	Mass [Da]		Sequence coverage [%]	Sequence range	Sequence
	Theoretical	Measured			
**A** Classical in-gel protein digestion	1120.45	1120.46	49	77–85	K.WENGECAQK.K
	915.46	915.47		100–107	K.IDALNENK.V
	1064.56	1064.58		108–116	K.VLVLDTDYK.K
	1192.66	1192.67		108–117	K.VLVLDTDYKK.Y
	2817.23	2817.26		118–140	K.YLLFCMENSAEPEQSLACQCLVR.T
	2833.21	2833.25		118–140	K.YLLFCMENSAEPEQSLACQCLVR.T + Oxidation (M)
	1244.57	1244.58		141–151	R.TPEVDDEALEK.F
	1634.76	1634.77		141–154	R.TPEVDDEALEKFDK.A
	2845.26	2845.47		155–178	K.ALKALPMHIRLSFNPTQLEEQCHI.-
	2861.26	2861.47		155–178	K.ALKALPMHIRLSFNPTQLEEQCHI.- + Oxidation (M)
	836.46	836.47		158–164	K.ALPMHIR.L
	852.46	852.46		158–164	K.ALPMHIR.L + Oxidation (M)
	1714.79	1714.80		165–178	R.LSFNPTQLEEQCHI.-
**B** Classical in-gel protein digestion and ZipTip	2312.20	2312.25	60	57–76	R.VYVEELKPTPEGDLEILLQK.W
	1120.44	1120.46		77–85	K.WENGECAQK.K
	902.55	902.56		92–99	K.TKIPAVFK.I
	1064.56	1064.58		108–116	K.VLVLDTDYK.K
	1192.66	1192.67		108–117	K.VLVLDTDYKK.Y
	2817.21	2817.26		118–140	K.YLLFCMENSAEPEQSLACQCLVR.T
	2833.18	2833.25		118–140	K.YLLFCMENSAEPEQSLACQCLVR.T + Oxidation (M)
	1244.56	1244.58		141–151	R.TPEVDDEALEK.F
	1634.74	1634.77		141–154	R.TPEVDDEALEKFDK.A
	2845.24	2845.47		155–178	K.ALKALPMHIRLSFNPTQLEEQCHI.-
	2861.22	2861.47		155–178	K.ALKALPMHIRLSFNPTQLEEQCHI.- + Oxidation (M)
	836.46	836.47		158–164	K.ALPMHIR.L
	852.46	852.46		158–164	K.ALPMHIR.L + Oxidation (M)
	1714.77	1714.80		165–178	R.LSFNPTQLEEQCHI.-

**C** μ-IMER	2706.23	2706.37	65	31–56	K.VAGTWYSLAMAASDISLLDAQSAPLR.V
	2722.23	2722.36		31–56	K.VAGTWYSLAMAASDISLLDAQSAPLR.V + Oxidation (M)
	2312.14	2312.25		57–76	R.VYVEELKPTPEGDLEILLQK.W
	1192.60	1192.67		108–117	K.VLVLDTDYKK.Y
	2817.13	2817.26		118–140	K.YLLFCMENSAEPEQSLACQCLVR.T
	1634.70	1634.77		141–154	R.TPEVDDEALEKFDK.A
	2845.18	2845.47		155–178	K.ALKALPMHIRLSFNPTQLEEQCHI.-
	2861.18	2861.47		155–178	K.ALKALPMHIRLSFNPTQLEEQCHI.- + Oxidation (M)
	836.36	836.47		158–164	K.ALPMHIR.L
	1714.72	1714.80		165–178	R.LSFNPTQLEEQCHI.-
**D** μ-IMER and ZipTip	2706.39	2706.37	80	31–56	K.VAGTWYSLAMAASDISLLDAQSAPLR.V
	2312.27	2312.25		57–76	R.VYVEELKPTPEGDLEILLQK.W
	1120.52	1120.46		77–85	K.WENGECAQK.K
	1248.60	1248.56		77–86	K.WENGECAQKK.I
	902.61	902.56		92–99	K.TKIPAVFK.I
	915.53	915.47		100–107	K.IDALNENK.V
	1192.73	1192.67		108–117	K.VLVLDTDYKK.Y
	2817.29	2817.26		118–140	K.YLLFCMENSAEPEQSLACQCLVR.T
	1244.63	1244.58		141–151	R.TPEVDDEALEK.F
	1634.80	1634.77		141–154	R.TPEVDDEALEKFDK.A
	2845.33	2845.47		155–178	K.ALKALPMHIRLSFNPTQLEEQCHI.-
	836.53	836.47		158–164	K.ALPMHIR.L
	852.53	852.46		158–164	K.ALPMHIR.L + Oxidation (M)
	1714.83	1714.80		165–178	R.LSFNPTQLEEQCHI.-
